# MiR-203 downregulation is responsible for chemoresistance in human glioblastoma by promoting epithelial-mesenchymal transition via SNAI2

**DOI:** 10.18632/oncotarget.3563

**Published:** 2015-03-12

**Authors:** Hongzhan Liao, Yifeng Bai, Shengcong Qiu, Lei Zheng, Lianyan Huang, Tianzhu Liu, Xin Wang, Yanting Liu, Ningbo Xu, Xiaohui Yan, Hongbo Guo

**Affiliations:** ^1^ Department of Neurosurgery, Neurosurgery Institute of Guangdong, Key Laboratory on Brain Function Repair and Regeneration of Guangdong, Zhujiang Hospital, Southern Medical University, Guangzhou, China; ^2^ Department of Oncology, Sichuan Academy of Medical Sciences & Sichuan Provincial People's Hospital, Chengdu, China; ^3^ Department of Laboratory Medicine, Nanfang Hospital, Southern Medical University, Guangzhou, China; ^4^ School of Public Health and Tropical Medicine, Southern Medical University, Guangzhou, China; ^5^ Research Center of Clinical Medicine, Nanfang Hospital, Southern Medical University, Guangzhou, China

**Keywords:** chemotherapy resistance, epithelial-mesenchymal transition, microRNAs, glioblastoma, SNAI2

## Abstract

Epithelial-mesenchymal transition (EMT) has been recognized as a key element of cell migration, invasion, and drug resistance in several types of cancer. In this study, our aim was to clarify microRNAs (miRNAs)-related mechanisms underlying EMT followed by acquired resistance to chemotherapy in glioblastoma (GBM). We used multiple methods to achieve our goal including microarray analysis, qRT-PCR, western blotting analysis, loss/gain-of-function analysis, luciferase assays, drug sensitivity assays, wound-healing assay and invasion assay. We found that miR-203 expression was significantly lower in imatinib-resistant GBM cells (U251AR, U87AR) that underwent EMT than in their parental cells (U251, U87). Ectopic expression of miR-203 with miRNA mimics effectively reversed EMT in U251AR and U87AR cells, and sensitized them to chemotherapy, whereas inhibition of miR-203 in the sensitive lines with antisense oligonucleotides induced EMT and conferred chemoresistance. SNAI2 was identified as a direct target gene of miR-203. The knockdown of SNAI2 by short hairpin RNA (shRNA) inhibited EMT and drug resistance. In GBM patients, miR-203 expression was inversely related to SNAI2 expression, and those tumors with low expression of miR-203 experienced poorer clinical outcomes. Our findings indicate that re-expression of miR-203 or targeting SNAI2 might serve as potential therapeutic approaches to overcome chemotherapy resistance in GBM.

## INTRODUCTION

Glioblastoma (GBM) is the most common primary malignant brain tumor in adults, with a median survival of approximately 14 months after diagnosis [1]. As one of the standard therapeutic approaches, chemotherapy is effective to reduce tumor size, inhibit distant metastasis and prolong patient survival [2]. However, GBM exhibits a high resistance to chemotherapy and recurrence is virtually assured [3]. Emerging evidence indicates a strong link between resistance to chemotherapy and the induction of epithelial-mesenchymal transition (EMT) in cancer [4]. EMT is a process during which cells undergo morphologic changes from epithelial phenotype to mesenchymal phenotype, resulting in enhanced motility and increased invasion, proliferation, and resistance to apoptosis [5, 6]. Tumor cells that have undergone EMT leave the primary tumor site, invade the extracellular matrix and basement membrane, colonize distant organs and form metastases. Therefore, determining the mechanisms that connect EMT and the development of drug resistance is essential for development of novel therapeutic strategies to overcome drug resistance.

MicroRNAs (miRNAs) are small non-coding RNAs that participate in many cellular processes as essential gene regulators. They modulate protein expression by promoting RNA degradation and inhibiting transcription after binding to the 3′-untranslated region (3′-UTR) of mRNA [7]. Accumulating evidence has demonstrated that miRNAs have a key role in drug resistance and EMT. For example, miR-27a is reported to reverse cisplatin resistance on bladder cancer cells by targeting SLC7A11 [8]. MiR-135a modulates paclitaxel resistance by targeting APC in human non-small cell lung cancer cells [9]. Moreover, recent study has shown that members of the miR-200 family play key roles in mediating the effects of TGF-β and other EMT regulators on EMT in breast cancer and lung adenocarcinoma cells [10, 11]. However, miRNAs and their target genes involved in EMT, resulting in resistance to chemotherapy, are still not fully understood.

In this study, we screened for miRNAs that were differentially expressed in the imatinib-resistant GBM cells, and identified that miR-203 was significantly downregulated. Next we explored the roles of miR-203 and its target SNAI2 in regulating EMT and drug resistance in GBM cells. Finally, we correlated the expression of miR-203 with the clinicopathological status and prognosis of GBM patients.

## RESULTS

### Imatinib-resistant U251AR and U87AR cells exhibit EMT characteristics

**Figure 1:**
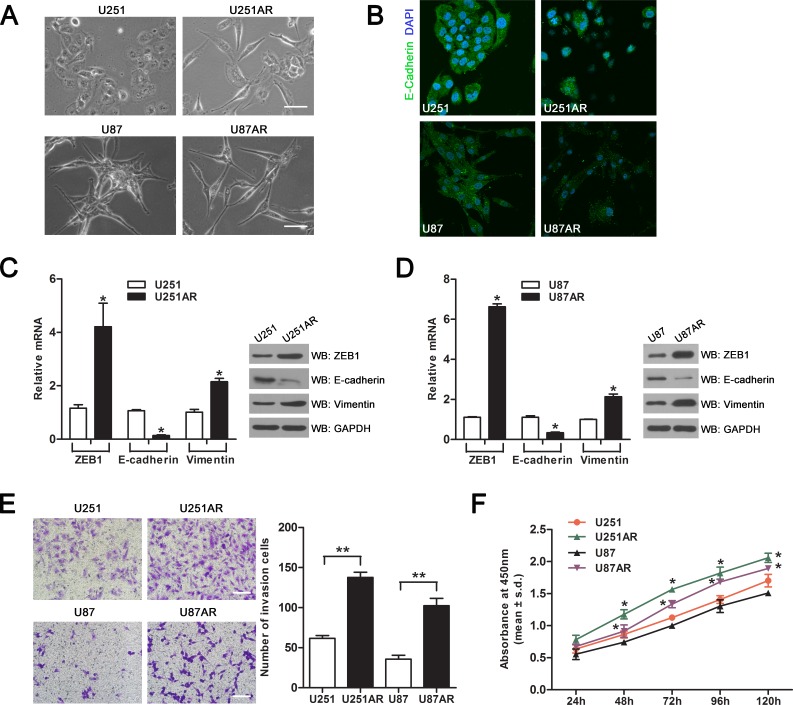
Imatinib-resistant U251AR and U87AR cells exhibit EMT characteristics (A) Morphological differences between parental cells and imatinib-resistant GBM cells. Scale bar, 100 μm. (B) Immunofluorescence staining of cell-cell junction protein E-cadherin. Parental and resistant GBM cells were stained with E-cadherin (green) as well as DAPI (blue) and pictures were taken at ×40 magnification. Nuclei are stained in blue with DAPI. (C) The mRNA and protein levels of EMT markers in U251 and U251AR cells were respectively detected by qRT-PCR and western blotting. (D) The mRNA and protein levels of EMT markers in U87 and U87AR cells were respectively detected by qRT-PCR and western blotting. (E) Transwell invasion assay proves an altered invasive behavior of imatinib-resistant GBM cells. Scale bar, 200 μm. (F) The cell viability of parental and resistant GBM cells after treatment with 50 μg/ml TMZ for 24, 48, 72, 96 and 120 h. Data are represented as mean±s.d. of three independent experiments. TMZ, temozolomide. *P < 0.05, **P < 0.001.

By using the parental cell lines U251 and U87, we previously established the imatinib-resistant GBM cell lines U251AR and U87AR [12], which had a cross-resistance to other anticancer drugs (etoposide/VP-16 and temozolomide/TMZ). We investigated whether the acquisition of the multidrug-resistant phenotype was accompanied by morphological changes of the cells. The parent U251 and U87 cells grew in clusters with tight cell-cell junctions, while U251AR and U87AR cells separated from one other and grew as loosely packed spindle-like cells (Figure 1A). This suggested U251AR and U87AR cells had undergone EMT resulting in the acquisition of mesenchymal properties. We next investigated the expression and localization of a key epithelial marker (E-cadherin) in the imatinib-resistant GBM cells compared with their parental cells. E-cadherin predominantly localized at cell-cell contacts in U251 and U87 cells, while the staining intensity was reduced in U251AR and U87AR cells (Figure 1B). Moreover, E-cadherin expression was significantly reduced at the mRNA and protein levels in U251AR and U87AR cells as compared with their parental cells (Figure 1C, D). We also examined the expression of other EMT marker genes by performing gene expression profiling in both U87 and U87AR cells. The gene expression profiling revealed that a range of epithelial marker genes were downregulated and many mesenchymal marker genes were upregulated in U87AR cells (Supplementary Table 1). Additionally, qRT-PCR and western blotting analysis showed that mesenchymal genes ZEB1 and vimentin were also upregulated in U251AR and U87AR cells (Figure 1C, D).

To examine whether EMT can promote cell invasion, we next performed a cell invasion assay which observed a significant increase in the invasive capacity of imatinib-resistant cells compared with their parental cells (Figure 1E). Furthermore, cell viability assay showed that resistant GBM cells were significantly more capable of growth than their parental cells (Figure 1F). All together, these data indicate that imatinib-resistant U251AR and U87AR cells have undergone EMT with enhanced invasiveness and increased cell viability.

### miR-203 is downregulated in imatinib-resistant GBM cells and its re-expression sensitizes cells to anticancer drugs and reverses EMT-like properties

**Figure 2:**
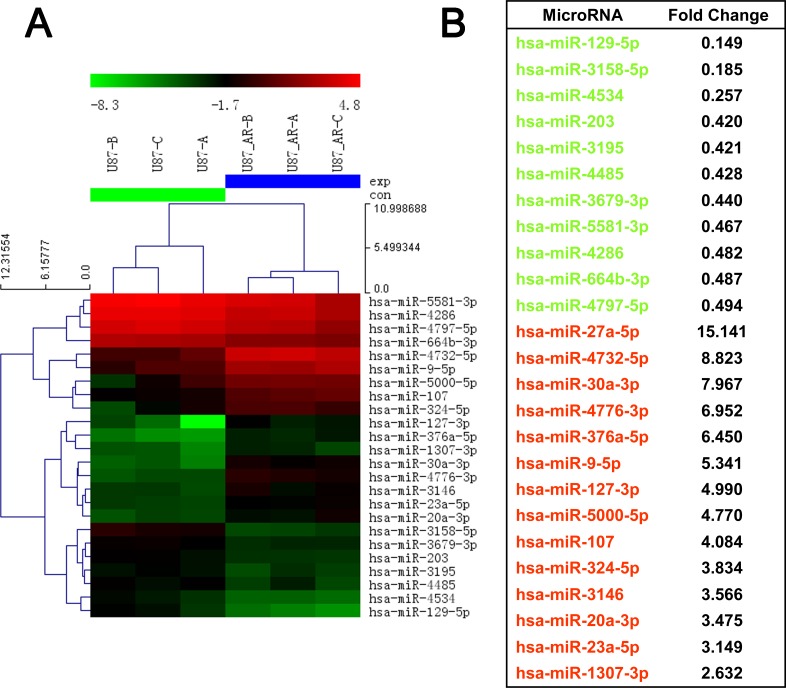
MicroRNA dysregulation in the imatinib resistant GBM cell line U87AR (A) Heatmap representation of differentially expressed microRNAs in the U87 and U87AR cells. Rows, miRNA; columns, independent biological replicates. Upregulated microRNAs are shown in red, while downregulated microRNAs are shown in green. (B) Differentially expressed microRNAs between U87 and U87AR cells.

To screen miRNAs that are potentially involved in the acquisition of drug resistance and induction of EMT, we performed microarray miRNA analysis on U87AR and its parental U87 cells. Microarray analysis revealed a significant downregulation of 11 miRNAs and upregulation of 14 miRNAs in U87AR compared with U87 cells (Figure 2A, B).

**Figure 3:**
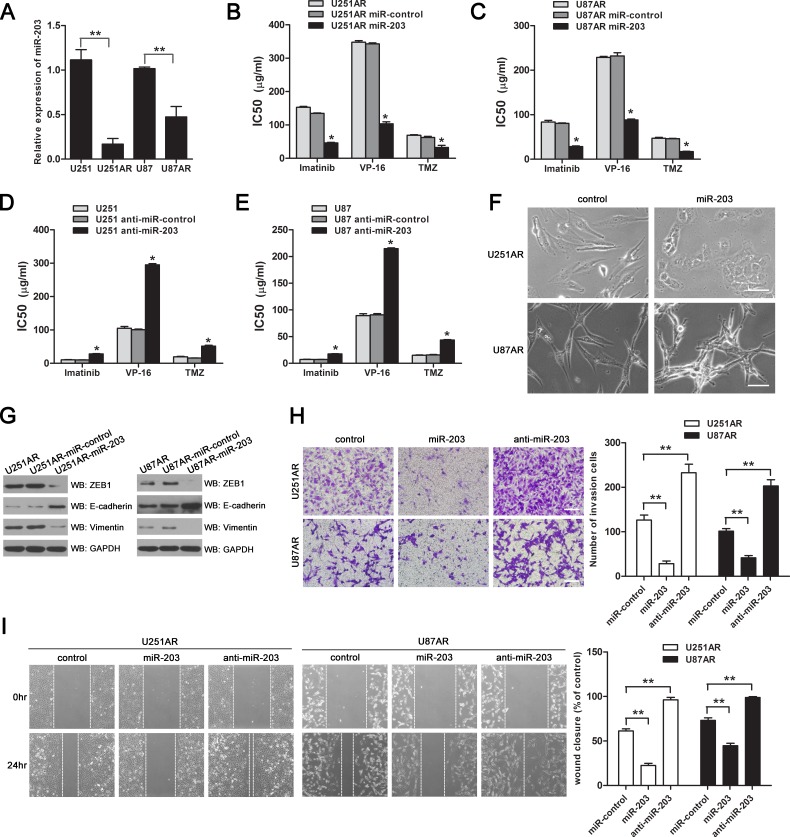
Re-expression of miR-203 in U251AR and U87AR cells sensitizes cells to anticancer drugs and reverses EMT while knockdown of miR-203 promotes resistance to anticancer drugs in U251 and U87 cells (A) qRT-PCR data validation of the downregulation of miR-203 in imatinib-resistant GBM cells compared with their parental cells, normalized to U6RNA, which was obtained from miRNA microarrays. (B, C) The sensitivities of U251AR and U87AR cells to imatinib, VP-16 and TMZ after transfected with miR-203 or miRNAs control. (D, E) Transfection with anti-miR-203 promotes resistance to imatinib, VP-16 and TMZ in U251 and U87 cells. (F) Morphology of U251AR and U87AR cells transfected with miRNA control or miR-203. Scale bar, 100 μm. (G) Western blotting show that re-expression of miR-203 modulates the expression of EMT markers. (H, I) U251AR and U87AR cells were transfected with miR-203 or anti-miR-203, and then collected for transwell invasion assay or wound healing assay. Shown were pictures of representative fields for each experiment. Scale bar, 200 μm. Data were expressed as mean±s.d. from three independent experiments. VP-16, etoposide; TMZ, temozolomide. *P < 0.05, **P < 0.01.

MiR-203 was among the top downregulated miRNAs in U87AR cells and its downregulation was further validated by qRT-PCR (Figure 3A). To explore the potential role of miR-203 in drug resistance and EMT, we used a miR-203 mimic (miR-203) and antagomir-203 (anti-miR-203) to modulate cellular levels of miR-203 in GBM cells. Expression of miR-203 was determined by qRT-PCR assay after miR-203 or anti-miR-203 was successfully transferred into U251AR or U87 cells, respectively (Supplementary Figure 1A, B).

The half maximal inhibitory concentrations (IC50) values of anticancer drugs (imatinib, VP-16 and TMZ) in the imatinib-resistant cells and their parental cells transfected with miR-203 or anti-miR-203 were determined by cell counting kit-8 (CCK-8) assay to test the effect of miR-203 expression on the sensitivities of GBM cells to imatinib, VP-16 and TMZ. As shown in Figure 3B, C, the IC50 values of imatinib, VP-16 and TMZ in the U251AR and U87AR cells transfected with miR-203 were significantly decreased by 1.9-3.3-fold, suggesting that upregulation of miR-203 expression could enhance the sensitivities of U251AR and U87AR cells to all the three anticancer drugs. In contrast, the IC50 values of imatinib, VP-16 and TMZ in the U251 and U87 cells transfected with anti-miR-203 were increased by 2.4-3.2-fold (Figure 3D, E), indicating that loss of miR-203 promotes resistance to anticancer drugs.

Next, we asked whether miR-203 re-expression could reverse EMT-like properties of the imatinib-resistant GBM cells. As shown in Figure 3F, miR-203-transfected U251AR and U87AR cells showed epithelial cell features, characterized by cellular aggregation. Western blotting analysis showed that miR-203 significantly increased the expression of epithelial marker E-cadherin while decreased that of mesenchymal markers ZEB1 and vimentin in miR-203-transfected U251AR and U87AR cells (Figure 3G). Furthermore, both transwell invasion and “wound healing” assays showed decreased invasion and migration activity of U251AR and U87AR cells in the presence of miR-203, but remarkably enhanced invasion and migration activity upon anti-miR-203 treatment (Figure 3H, I). Additionally, we found that miR-203 re-expression could induce cell apoptosis in U251AR and U87AR cells (Supplementary Figure 2).

Finally, to determine the potential clinicopathological implications of altered miR-203 expression, we examined the expression levels of miR-203 in the specimens from 80 patients with glioma by qRT-PCR. The relationships between clinicopathologic characteristics and miR-203 expression levels in patients with glioma are summarized in Table 1. No significant association between miR-203 expression level and patient's sex or age was observed in any of the 80 glioma cases. However, the expression of miR-203 was positively correlated with the tumor grading (WHO I-II vs. WHO III-IV) (*P*=0.004, Mann-Whitney test) in glioma patients (Table 1).

**Table 1:** Associations between miR-203 expression levels and clinicopathologic characteristics in 80 patients with glioma

Characteristics	No.	%	Median expression of miR-203/U6	*P*
Gender				
Male	46	57.5	0.480	0.282
Female	34	42.5	0.294	
Age, year				
<50	37	46.3	0.403	0.323
≥50	43	53.7	0.476	
WHO Grade				
I / II	22	27.5	0.675	0.004*
III / IV	58	72.5	0.257	

Abbreviation: miR = microRNA; WHO = World Health Organisation.

* Statistical significance (*p* < 0.05).

### SNAI2 is a direct target of miR-203

**Figure 4:**
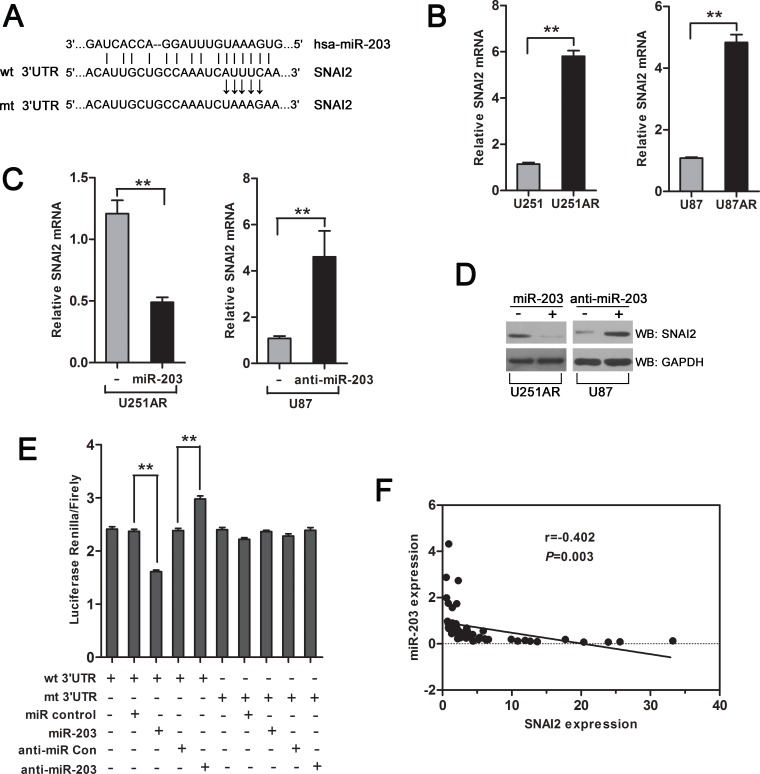
miR-203 targets SNAI2 (A) Sequence alignment of human miR-203 with 3′-UTR of SNAI2. The seed sequence of miR-203 (top) matches 3′-UTR of SNAI2 (middle). Bottom, mutation of the 3′-UTR of SNAI2 in mutant luciferase reporter construct. (B) qRT-PCR shows that SNAI2 is upregulated in U251AR and U87AR cells. (C, D) qRT-PCR and western blotting assay of U251AR or U87 cells transfected with miR-203 or anti-miR-203, respectively. (E) Dual luciferase assay was performed in U251AR cells transfected with luciferase construct alone or cotransfected with miR-203 and anti-miR-203. Firefly luciferase construct containing mutant target site of the SNAI2 3′-UTR was generated and transfected as indicated. Firefly luciferase activity was normalised to Renilla luciferase activity for each sample. (F) Pearson correlation analysis shows a significant inverse correlation between miR-203 expression level and SNAI2 mRNA level in human GBM specimens. The results shown represent the mean±s.d. from three independent experiments. **P < 0.01.

We next explored the molecular mechanisms responsible for the drug resistance and EMT-suppressive effect of miR-203. The predicted target genes of miR-203 were retrieved from miRTarBase and TargetScan databases. SNAI2 (also known as slug), a transcriptional repressor of E-cadherin, was one of the potential candidates, which gained our attention, because of the importance of SNAI2 in EMT and drug resistance [13, 14] and the high conservation of the putative miR-203-binding sequences in the SNAI2 3′-UTR (Figure 4A). Furthermore, we verified that SNAI2 mRNA was upregulated in U251AR and U87AR cells (Figure 4B). To investigate whether SNAI2 is regulated by miR-203, we next transfected cells with miR-203 or anti-miR-203 and examined SNAI2 expression. qRT-PCR and western blotting analysis demonstrated that, in U251AR cells with high levels of SNAI2, restoration of miR-203 reduced the mRNA and protein expression of SNAI2 (Figure 4C, D). In contrast, in U87 cells, which express low levels of SNAI2, miR-203 inhibition by transfection with anti-miR-203 increased the mRNA and protein levels of SNAI2 (Figure 4C, D). These results demonstrate that miR-203 directly interacts with SNAI2 mRNA and represses its expression.

To assess whether miR-203 directly regulates SNAI2 expression through the target site in the 3′-UTR of SNAI2, reporter constructs containing either the wild-type (WT) SNAI2 3′-UTR or SNAI2 3′-UTR with mutation at the predicted miR-203 target sequence were cotransfected into U251AR cells together with miR-203, miRNA control, anti-miR-203 or anti-miRNA control. Transduction of miR-203 caused marked inhibition of the WT SNAI2 3′-UTR, but had no effect on mutant SNAI2 3′-UTR (Figure 4E). Meanwhile, miR-203 inhibition by anti-miR-203 substantially increased luciferase activities of WT SNAI2 3′-UTR compared with anti-miRNA control (Figure 4E). All these results strongly suggest that SNAI2 is a direct target of miR-203 in GBM cells.

Finally, to examine the pathological relevance of this interaction, we detected the expressions of miR-203 and its target SNAI2 in human GBM tissues. As shown in Figure 4F, SNAI2 expression was negatively correlated with miR-203 expression in clinical GBM samples (Pearson's correlation *r*= −0.402, *p*=0.003). In summary, SNAI2 is upregulated in the imatinib-resistant GBM cells and a direct target of miR-203, and their expression is negatively correlated in GBM patients.

### Loss of SNAI2 restores sensitivity to anticancer drugs and reduces the invasion and migration capacity of U251AR cells

**Figure 5:**
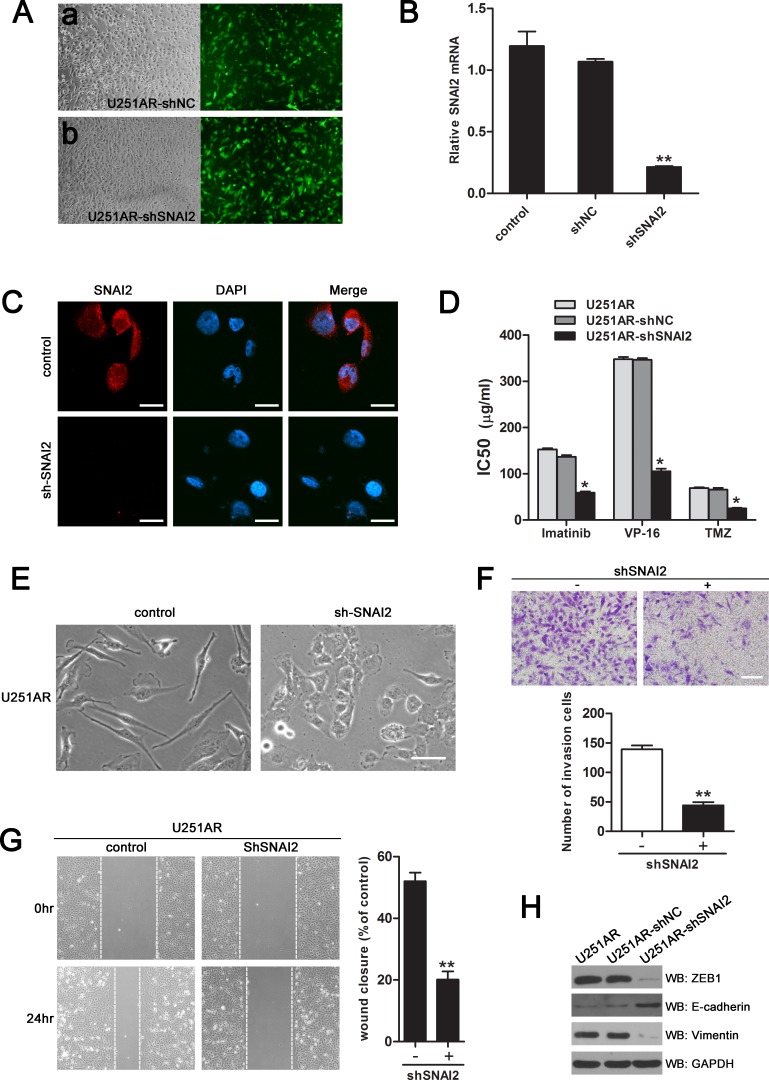
Silencing of SNAI2 phenocopies the effects of miR-203 re-expression on sensitization of U251AR cells to anticancer drugs and reversion of EMT (A) a. U251AR/shNC cells; b. U251AR/shSNAI2 cells. Light microscopy, 100× (a, b); Fluorescent microscopy, 100× (a, b). shSNAI2 and negative vector (shNC) were transfected into U251AR cells. At 48 h after transfection, fluorescent microscopy showed emission green fluorescence. (B) qRT-PCR validate the downregulation of SNAI2 after shRNA knockdown in U251AR cells. (C) Immunofluorescence analysis of the endogenous SNAI2 protein (red, left panels) in U251AR cells transfected with shSNAI2 or negative vector. Nuclei are stained in blue with DAPI. Scale bar, 20 μm. (D) The sensitivities of U251AR and U251AR/shSNAI2 to different concentrations of TMZ, imatinib and VP-16. (E) Morphology of U251AR cells transfected with negative vector or shSNAI2 vector. Scale bar, 100 μm. (F) SNAI2 knockdown reduces the invasion capacity of U251AR cells. Scale bar, 200 μm. (G) U251AR cell monolayer was transfected as indicated and scratched, then the migration of the cells towards the wound was visualised. Images were taken at various time points and Image J was used to determine the migration distance. (H) Western blotting show that silencing of SNAI2 can modulate the expression of EMT markers. VP-16, etoposide; TMZ, temozolomide. Data are presented as mean±s.d. of three independent experiments. *P < 0.05, **P < 0.01.

The direct targeting of SNAI2 by miR-203 led us to hypothesize that downregulation of SNAI2 by miR-203 in resistant GBM cells could be involved in drug resistance and/or EMT. For this purpose, we performed shRNA-mediated knockdown of SNAI2 in U251AR cells. The negative vector (shNC) and shSNAI2 were transfected into U251AR cells. At 48 hours post-transfection, fluorescent microscopy showed emission of green fluorescence (Figure 5A). qRT-PCR and immunofluorescence analysis showed that SNAI2 expression level was significantly decreased in shSNAI2-transfected U251AR cells, compared to control cells (Figure 5B, C). Knockdown of SNAI2 could indeed partially phenocopy the effects observed on sensitization to anticancer drugs upon overexpression of miR-203 (Figure 5D). Silencing of SNAI2 led to significant changes in cell morphology. The scattered, mesenchymal-like U251AR cells began to exhibit a more epithelial-like cobblestone appearance (Figure 5E). As shown in Figure 5F, G, the invasion and migration of U251AR cells were markedly reduced after the inhibition of SNAI2 expression. Moreover, we examined the expression of those EMT marker that we had analyzed upon overexpression of miR-203 and also after knockdown of SNAI2 (Figure 5H). Silencing of SNAI2 increased E-cadherin expression and decreased the mesenchymal markers ZEB1 and vimentin, showing that the involvement of miR-203 in EMT could, at least in part, be via targeting SNAI2.

### SNAI2 contributes to chemoresistance and EMT in GBM cells

**Figure 6:**
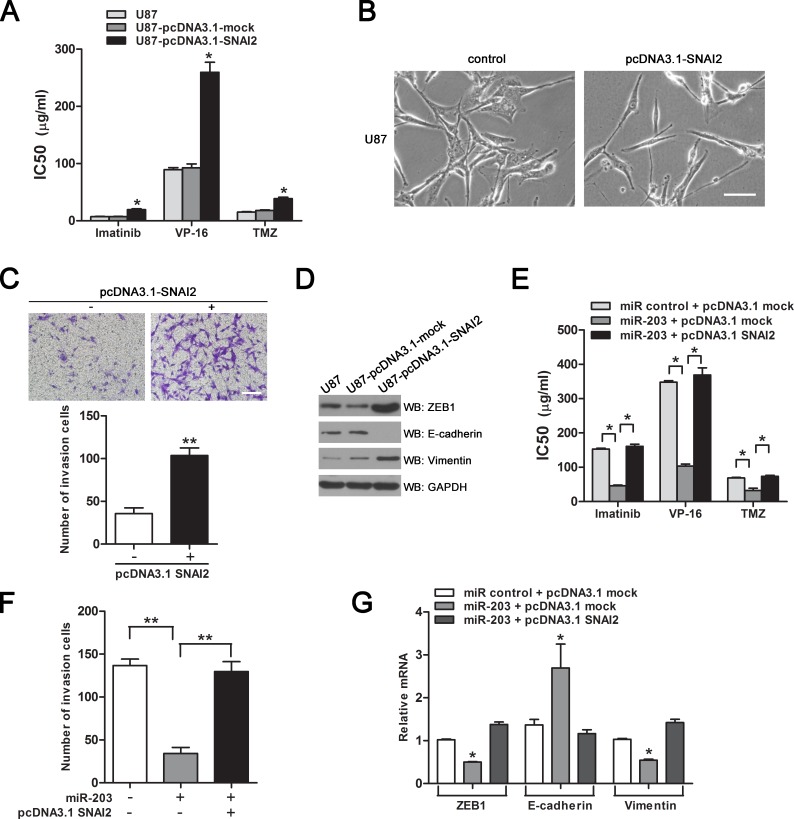
SNAI2 contributes to chemoresistance and EMT in GBM cells (A) Overexpression of SNAI2 promotes resistance to imatinib, VP-16 and TMZ. (B) Morphology of U87 cells transfected with pcDNA3.1-mock or pcDNA3.1-SNAI2. Scale bar, 100 μm. (C) Invasion of U87 cells after pcDNA3.1-SNAI2 transfection. Scale bar, 200 μm. (D) Protein expression of EMT markers in U87 cells transfected with pcDNA3.1-mock or pcDNA3.1-SNAI2, determined by western blotting. (E) The sensitivities to imatinib, VP-16 and TMZ were measured after cells transfected with indicated constructs and miR-203 in U251AR. (F) Invasion assay of U251AR cells expressing indicated vectors and miR-203. (G) qRT-PCR for EMT markers in U251AR cells expressing indicated constructs and miR-203. *P < 0.05, **P < 0.01.

We asked if overexpression of SNAI2 could induce drug resistance and EMT in parental GBM cells. For this purpose, we developed a stably SNAI2 over-expressing U87-pcDNA3.1-SNAI2 subline by transfection with pcDNA3.1-SNAI2 (Supplementary Figure 3). As shown in Figure 6A, B, overexpression of SNAI2 reduced the sensitivity of U87 cells to anticancer drugs and induced a shift in cell morphology from tight cell-cell junctions to loss of cell-to-cell contact. Also, enforced expression of SNAI2 promoted cell invasion *in vitro* (Figure 6C). Furthermore, the downregulation of epithelial marker E-cadherin and the upregulation of mesenchymal markers ZEB1 and vimentin were observed in pcDNA3.1-SNAI2-transfected U87 cells (Figure 6D).

To further define the involvement of SNAI2 in the suppression of chemoresistance and EMT by miR-203, SNAI2 was transfected into miR-203-overexpressing U251AR cells. Next, we performed drug sensitivity assay to evaluate the chemoresistance changes in these cells. Ectopic expression of SNAI2 significantly rescued miR-203-induced inhibition of drug resistance (Figure 6E). Moreover, reintroduction of SNAI2 markedly antagonized the inhibitory effect of miR-203 on cell invasion (Figure 6F), abolished the mRNA expression of E-cadherin and restored ZEB1 and vimentin expression (Figure 6G). These data suggest a crucial role of SNAI2 in driving chemoresistance and EMT of GBM cells.

### Low expression of miR-203 in GBM is associated with chemotherapeutic resistance and poor patient prognosis

**Figure 7:**
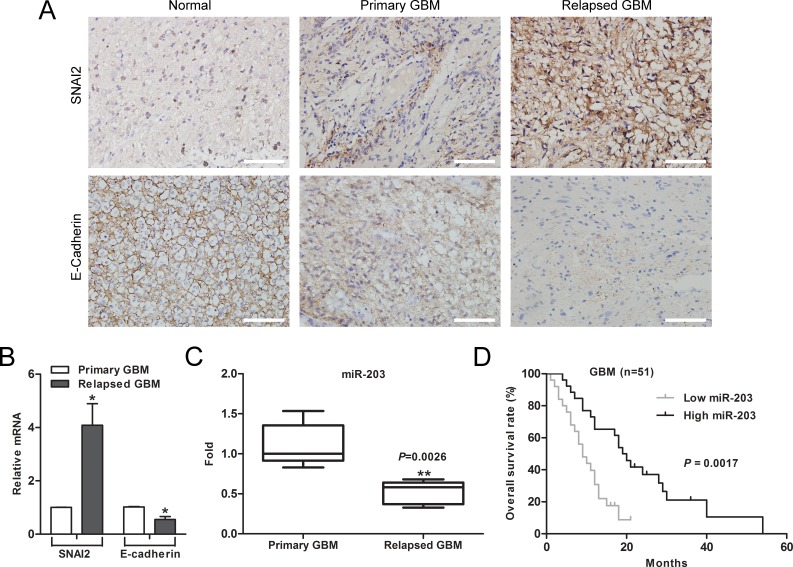
Downregulation of miR-203 correlates with chemotherapy resistance and poor patient survival in GBM (A) Expression of SNAI2 and E-cadherin in resected human GBM specimen was assessed by immunohistochemistry assay. Scale bar, 100 μm. (B) Average expression levels of SNAI2 and E-cadherin in human primary GBM specimens and relapsed GBM tissues. (C) The expression of miR-203 was significantly reduced in relapsed GBM patients. (D) Kaplan-Meier overall survival curve according to miR-203 expression levels in GBM patients (*p* = 0.0017). *P < 0.05, **P < 0.01.

To further evaluated the clinical significance of miR-203 expression in chemotherapeutic resistance and patient prognosis of GBM, SNAI2 expression was detected in tissues from 35 cases of patients with primary GBM and 16 cases of patients with relapsed GBM by immunohistochemistry. We found that the expression level of SNAI2 in relapsed GBM patients with treatment of temozolomide for 6 months was higher than that in primary GBM patients without treatment of temozolomide (Figure 7A). In contrast, E-cadherin was lowly expressed in the relapsed GBM patients (Figure 7A). Furthermore, qRT-PCR showed that the mRNA level of SNAI2 was significantly increased in relapsed GBM samples, whereas E-cadherin mRNA level was reduced compared to primary GBM tissues (Figure 7B). Finally, we found that the expression of miR-203 was significantly reduced (*p*=0.0026) in relapsed GBM tissues (Figure 7C). Moreover, patients with higher expression levels of miR-203 survived longer (*p* = 0.0017) than patients with lower expression levels (Figure 7D).

## DISCUSSION

In this study, we demonstrated that the imatinib-resistant U251AR and U87AR cells underwent EMT, and miR-203 was downregulated in these cells and clinical relapsed GBM specimens. Re-expression of miR-203 was capable not only of reversing EMT but also of sensitizing cells to anticancer drugs and reducing invasion and migration. Moreover, miR-203 suppressed the EMT and chemoresistance of GBM cells by targeting SNAI2. Our findings suggest that after developing drug resistance, miR-203 expression is reduced leading to a higher expression of SNAI2 and other targets, and the cells become more mesenchymal-like and invasive. These results are supported by clinical data where we found an inverse correlation between the expression of miR-203 and its target SNAI2 in GBM samples. Importantly, the significance and clinical relevance of miR-203 were further demonstrated in GBM patients.

Consistent with our finding, increasing evidence demonstrates that miRNAs are associated with drug resistance and EMT in many types of tumors. Ujifuku *et al.* [15] showed that miR-195, miR-455-3p and miR-10a upregulated in temozolomide (TMZ)-resistant GBM cells, played a critical role in acquired TMZ resistance. Similarly, downregulation of miR-181 was responsible for resistance to imatinib by directly targeting the Bcl-2 family member Mcl-1 in chronic myelogenous leukemia cells [16]. Also, miR-221 and miR-222 were upregulated while miR-21, miR-342, and miR-489 were downregulated in tamoxifen-resistant MCF-7 cells; the reintroduction of miR-221 or miR-222 rendered the parent MCF-7 cells resistance to tamoxifen through inhibiting their target p27Kip1, which was reduced by 50% in resistant cells [17]. However, not many reports [18-21] explored the involvement of miRNAs in the development of multidrug resistance in cancer. Thus, it is a great strength of our present study which demonstrated that miR-203 reversed the resistance of GBM cells to three different anticancer drugs. Although studies have investigated the drug resistance role of a number of miRNAs in various tumors, the overwhelming majority of other miRNAs are yet to be further studied regarding the drug resistance.

Numerous inducers of EMT in cancer cells have been identified including transforming growth factor-β, Wnt/β-catenin, Snail/SNAI2, Twist and talin [22]. Recently, gain of Twist1 and loss of E-cadherin observed in GBM specimens were correlated with shorter survival and poor temozolomide response in GBM patients, suggesting the involvement of EMT in GBM progression [23, 24]. Furthermore, acquisition of EMT features has been associated with drug resistance which could promote recurrence and metastasis after standard chemotherapeutic treatment [25]. In this paper, we demonstrate that EMT also occurs in the imatinib-resistant GBM cells. Similarly, others characterized EMT in tamoxifen-resistant breast cancer cells. Ward *et al.* [26] observed downregulation of epithelial marker (E-cadherin) expression that was accompanied by the upregulation of mesenchymal markers (TGFB2, SMAD3 and ZYX) and EMT transcription factor, SNAI2, in tamoxifen-resistant breast cancer cells compared with the parental cells. These findings support our own observations. Another report showed that EMT induction in anticancer drug-resistant cells was owing to increased levels of phosphorylated beta-catenin, which was associated with epidermal growth factor receptor (EGFR), leading to increased transcription of EMT regulators [27]. However, in brain tumor, the role of miRNAs in co-regulation of EMT and drug resistance remained largely unexplored. This led us to investigate the potential role of miRNAs in EMT followed by acquired resistance to chemotherapy in GBM.

Previous studies have shown that miR-203 is downregulated in metastatic prostate cancer [28] and colorectal carcinoma [29]. Furthermore, downregulation of miR-203 confers tumorigenicity ability to esophageal squamous cell carcinoma cells [30]. A recent study by Ding *et al.* [31] described the tumor suppressive effects of miRNA-203 in breast cancer, showing that loss of miRNA-203 led to increased invasion and metastatic potential of the cell system, similar to the results we have shown in GBM. However, other reports also suggest that elevated expression of miR-203 in pancreatic tumors is associated with poorer survival [32]. We found that miR-203 suppressed EMT and chemoresistance by targeting SNAI2 in GBM cells. These results indicate that miR-203 may have a dual function as both a tumor suppressor and an oncogene, depending on the cellular context and tumor type. A number of groups have reported DNA methylation-mediated downregulation of miRNAs by proximal CpG islands [33, 34]. Thus, DNA methylation may be involved in the regulation of miR-203 in imatinib resistant GBM cells.

Our present data further demonstrate that SNAI2 is a direct target of miR-203 and that miR-203-mediated inhibition of SNAI2 is dependent on a conversed motif in the 3′-UTR of SNAI2. Recent independent studies have shown that overexpression of SNAI2 alters cell invasion, motility, chemoresistance, metastasis and poor prognosis in several human cancers [35-38]. As a member of the snail family of transcription factors, SNAI2 can repress E-cadherin transcription and induce EMT directly [39]. Therefore, SNAI2 overexpression due to reduction of miR-203 may result in EMT and chemoresistance in GBM via these pathways. Additionally, miR-203 may relieve E-cadherin from transcriptional repression by targeting SNAI2 signaling. Nevertheless, because one single miRNA might have multiple targets, judicious considerations are essential for identiﬁcation of the main functional targets.

In summary, our study indicates that miR-203 is an effective inhibitor of EMT and chemoresistance, and consequently has an important role in the development of GBM. We further disclose that high expression of miR-203 predicts better prognosis in human GBM. Importantly, miR-203 may serve as an important molecular biomarker for the GBM patients with drug resistance problem and a target for treatment of this lethal disease.

## MATERIALS AND METHODS

### Patients and specimens

Patient tissue samples were obtained from 999 Brain Hospital (Guangzhou, China), Zhujiang and Nanfang Hospitals (Southern Medical University, Guangzhou, China). Patients enrolled in this study included 9 grade I astrocytoma cases, 13 grade II astrocytoma cases, 7 grade III astrocytoma cases, and 51 GBM cases. Among 51 GBM cases, 16 GBM cases were relapsed 6 months after temozolomide therapy. Tissue specimens were snap-frozen in the operating room immediately during surgery and sent to a pathology department for diagnosis by a board-certified neuropathologist. For each patient, a frozen tumor sample (stored at −80 °C) and a paraffin-embedded tissue specimen had to be available. Under the protocol approved by the Institutional Review Board, informed consents were obtained from the patients or their guardians according to institutional guidelines.

### Cell culture

Human GBM cell lines U251 and U87, were obtained as a gift from College of Public Health of Southern Medical University, GuangZhou, China. The stable imatinib-resistant lines, U251AR and U87AR, were established and maintained in our laboratory [12]. The cells were cultivated in Dulbecco's modified Eagle's medium (DMEM, Invitrogen) containing 10% fetal bovine serum (Invitrogen), penicillin (200 units/ml) and streptomycin (100 μg/ml), and were incubated at 37 °C in a humidified incubator with an atmosphere of 5% CO2. To maintain the multidrug resistant phenotype, U251AR and U87AR were alternately fed with drug-free medium and medium containing 122 μg/ml of imatinib.

### Cell transfection

Cells were transiently transfected with 100 nmol/l of miR-203 mimics (miR-203), antagomirs (anti-miR-203) and miRNA negative control, or 60 nmol/l short hairpin RNA (shRNA) specific to SNAI2, scrambled shRNA negative control (shNC) (Genepharma, Shanghai, China) by using Lipofectamine 2000 and OPTI-MEM I (Invitrogen). All of the RNA oligoribonucleotides were purchased from Genepharma (GenePharma, Shanghai, China) and the sequences are listed in Supplementary Table 2. For stable transfection, SNAI2 expression plasmid (pcDNA3.1 SNAI2) and empty plasmid (pcDNA3.1 mock) were transfected into GBM cells by using Lipofectamine 2000. Positive transfectants were selected in 500 μg/ml Geneticin (G418, Invitrogen). Individual colonies were harvested 24 h later for the evaluation of gene expression or functional assays.

### MiRNA microarray analysis

MiRNA microarray analysis was done in the parental and resistant U87 lines. Briefly, total RNA was isolated using TRIzol (Invitrogen) and the RNeasy mini kit (Qiagen) according to the manufacturer's instructions. The samples were labeled using the miRCURY^TM^ Hy3^TM^/Hy5^TM^ Power labeling kit (Exiqon) and hybridized on the miRCURY LNA Array (Exiqon, version 11.0). Scanning was performed with the Axon GenePix 4000B microarray scanner. GenePix pro version 6.0 was used to read the raw intensity of the image. Background subtraction and normalization were performed. We selected miRNAs whose expression levels between the parental and resistant U87 lines differed by at least 1.5-fold.

### RNA isolation, reverse transcription, and quantitative real-time PCR

Total RNA was extracted using Trizol reagent (Invitrogen) according to the manufacturer's protocol. To quantitate miR-203 expression, total RNA was polyadenylated and reversely transcribed using miRNAs qPCR Quantitation Kit (Genepharma, Shanghai, China). To measure the mRNA levels of SNAI2, ZEB1, E-cadherin and vimentin, total RNA was reversely transcribed using primeScript RT reagent Kit (Takala, Dalian, China). Quantitative real-time PCR was carried out in ABI7500 sequence detection system (Applied Biosystems, Foster City, CA, USA) using SYBR Green according to the manufacturer's instructions. The primers were listed in Supplementary Table 3. Glyceraldehyde-3-phosphate dehydrogenase (GAPDH) or U6 snRNA was used as an endogenous control. All samples were normalized to internal controls and fold changes were calculated through relative quantification (2^−ΔΔCt^).

### Western blot analysis

Protein lysates were separated by 10% SDS-PAGE, and electrophoretically transferred to PVDF (polyvinylidene difluoride) membrane (Millipore). Then, the membrane was incubated with rabbit monoclonal antibody against human SNAI2, ZEB1, E-cadherin and vimentin (Cell Signaling Technology, USA) followed by HRP (horseradish peroxidase)-labeled goat-anti rabbit IgG (Santa Cruz Biotechnology, USA) and detected by chemiluminescence. GAPDH was used as a protein-loading control.

### Luciferase reporter assay

We cloned the miR-203 response element (wide type or mutated) in the 3′-untranslated regions (3′-UTR) of SNAI2 into psiCheck2 plasmid downstream of luciferase reporter gene. Luciferase activities were measured using a luciferase assay kit (Promega, Madison, WI, USA), and target effect was expressed as relative luciferase activity of the reporter vector with target sequence over the one without target sequence.

### *In vitro* invasion assay

Cells growing in the log phase were treated with trypsin and re-suspended as single cell solutions. A total of 1×10^5^ cells were seeded on a fibronectin-coated polycarbonate membrane insert in a transwell apparatus (Corning Inc., USA). In the lower chamber, 600 μl DMEM with 10% FBS were added as a chemoattractant. After the cells were incubated for 48 h at 37 °C and 5% CO2 incubator, the insert was washed with PBS, and cells on top surface of the insert were removed by a cotton swab. Except the transwell membrane was precoated with ECMatrix and the cells were stained with crystal violet for 10 min and rinsed with PBS. The chambers were then photographed to compare the amount of invasive cells on the underside of the membrane for five predetermined fields (×200). All assays were independently repeated for at least three times.

### Wound healing assay and flow cytometric analysis of apoptosis

Cells were plated at a density of 5×10^5^/cm^2^ in a six-well plate, and incubated at 37 °C in a CO2 incubator for 12 h, allowing cells to completely adhere and spread on the six-well plate. The cells grew and were maintained in serum-free DMEM for more than 24 h to create a confluent monolayer. The confluent monolayer was scraped with a sterile toothpick and washed with PBS. Serum-free DMEM was then added and the width of the wound gaps were measured using NIH Image J analysis and normalized to the time 0 wounds for four independent experiments. Apoptosis after transfection treatment was examined by using the Annexin V/Propidium Iodide Detection Kit (KeyGEN) according to the manufacturer's instructions.

### *In vitro* drug sensitivity assay and cell viability analysis

Cells were plated in 96-well plates at 1×10^4^ cells per well after transient transfection or adherence of stable transfected cells. After 24 h, the cells were treated with different concentrations of imatinib (Novartis, Basel, Switzerland), etoposide (VP-16) (Sigma Chemical Co., St. Louis, MO) and temozolomide (TMZ) (Sigma Chemical Co., St. Louis, MO), each at four concentrations ranging from 50 to 200 μg/ml for 48 h. The range of drug concentrations were based on earlier studies and aimed at obtaining IC50 values both for highly sensitive and resistant cases. The absorbance at 450 nm was measured after incubation with 10 μl of CCK-8 reagent (Dojindo, Molecular Technologies, Dojindo, Japan) for four hours. After shaking for one min, the spectrophotometric absorbance of the samples was determined by using Ultra Multifunctional Microplate Reader (Tecan) at 450 nm. The assay was conducted in five replicate wells for each sample and three parallel experiments were performed. For cell viability analysis, cells were plated in 96-well plates at 2×10^3^ per well in a final volume of 100 μl. And cells were incubated with 50 μg/ml TMZ for 24, 48, 72, 96 and 120 h. Cell growth were determined with CCK-8 according to the manufacturer's instructions.

### Immunofluorescence staining

Immunofluorescence staining was done following the standard protocol. Briefly, cells were fixed with 4% paraformaldehyde, permeabilized in 0.5% Triton X-100, and blocked with 10% goat serum. The cells were then incubated overnight with specific primary antibody. After washing with PBS, the cells were incubated with fluorescence-conjugated secondary antibody for one hour. The slides were then washed with PBS and mounted with mounting medium containing Anti-fade reagent and 4′, 6-diamidino-2-phenylindole (DAPI). Cells were viewed under a fluorescence microscope.

### Immunohistochemical analysis

For immunohistochemical analyses of GBM, paraffin-embedded samples were sliced and mounted on microscopic slides. Rabbit monoclonal anti-SNAI2 and anti-E-cadherin antibodies (1:200 dilutions, Cell Signaling Technology, USA) were used as the primary antibodies. Heat-induced epitope was formed with a microwave in 10 mmol/l citric acid buffer at pH 7.2. The samples were incubated with the antibody overnight in the same buffer followed by incubation with biotinylated secondary antibody (1:500 dilutions, Santa Cruz Biotechnology, USA). The bound antibodies were visualized by the avidin biotinylated peroxidase complex methods and diaminobenzidine tetrachloride (Santa Cruz Biotechnology).

### Statistical analysis

All statistical analyses were performed using SPSS13.0 software (IBM Corporation, New York, NY, USA) and GraphPad Prism software 5.0 (GraphPad Software, Inc., San Diego, CA, USA). The results were presented as mean±s.d. of three replicate assays. Statistical analyses were performed using either an analysis of variance (ANOVA) or Student's *t*-test. And statistical analysis of tissue specimens was performed using the Mann-Whitney test to evaluate the significance of differences between groups. The relationship between SNAI2 and miR-203 expression was explored by Pearson correlation. Kaplan–Meier survival curves were generated to evaluate the correlation of miR-203 expression levels with survival rate. All *P*<0.05 was considered to indicate statistical significance.

## SUPPLEMENTARY MATERIALS, FIGURES AND TABLES


