# Supplementary material for: MiR-203 downregulation is responsible for chemoresistance in human glioblastoma by promoting epithelial-mesenchymal transition via SNAI2
Source: Oncotarget. 2015 Mar 12;6(11):8914–28. doi: 10.18632/oncotarget.3563 (PMC4496192; doi:10.18632/oncotarget.3563)
Supplement: Supplementary file 1 [file oncotarget-06-8914-s001.pdf]

MiR-203 downregulation is responsible for chemoresistance in human glioblastoma by promoting epithelial-mesenchymal transition via SNAI2

Supplementary Material

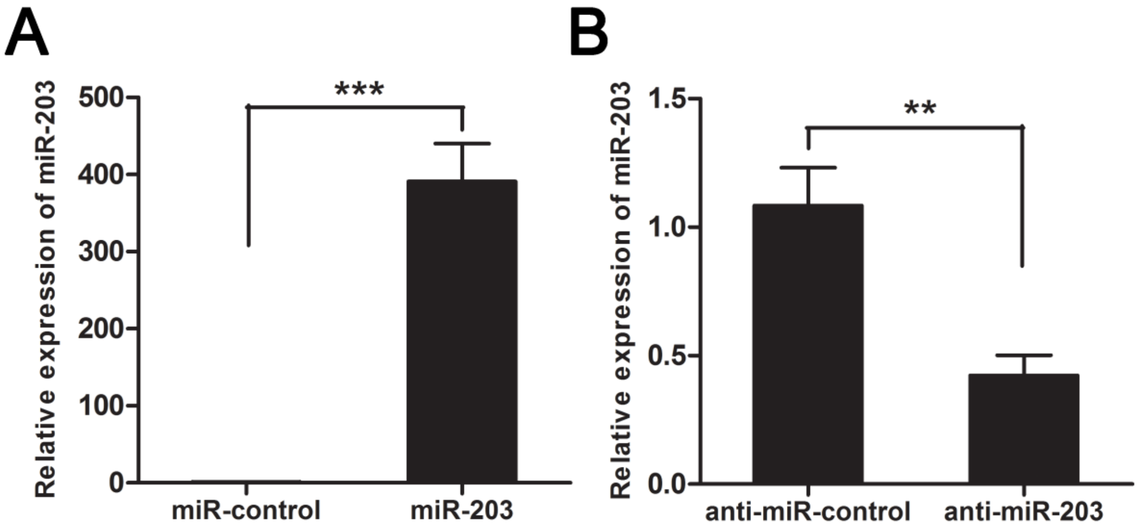

Supplementary Figure S1: The expression level of miR-203 in GBM cells after transfection. (A) qRT-PCR showing expression of miR-203 48hrs after transfection with control or miR-203. (B) qRT-PCR showing expression of miR-203 48hrs after transfection with control or anti-miR-203. $^{**}P < 0.001$, $^{***}P < 0.0001$.

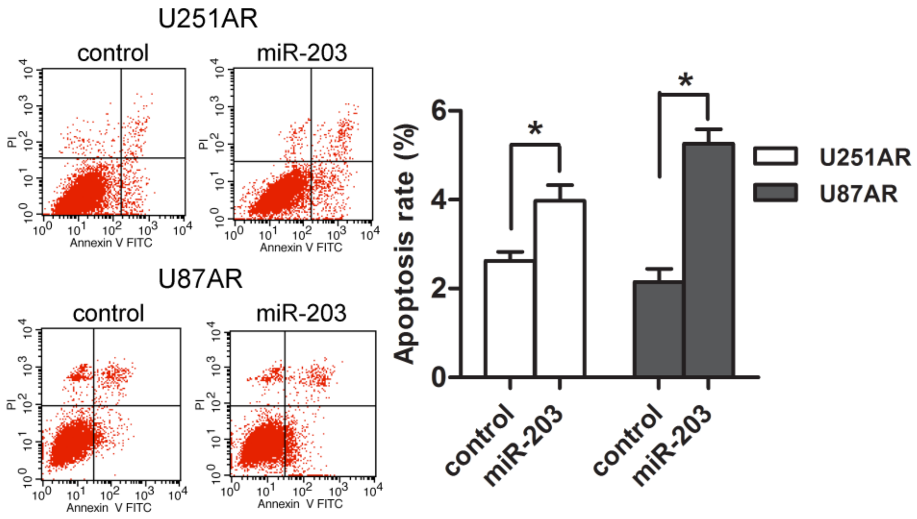

Supplementary Figure S2: U251AR and U87AR cells were transfected with control or miR-203 for 48 hours, and the apoptosis was measured by Annexin V staining and flow cytometry. *$P < 0.05$.

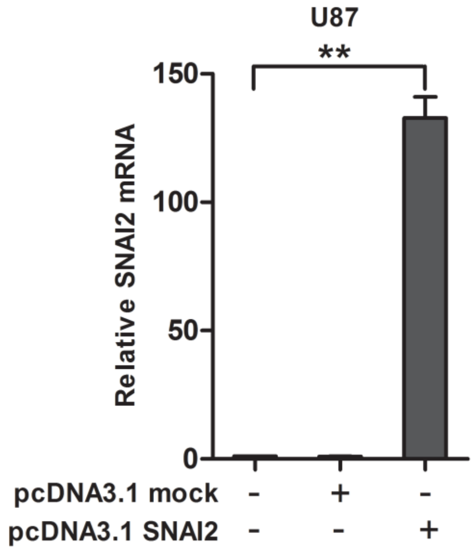

Supplementary Figure S3: SNAI2 mRNA level was assessed after transfection of pcDNA3.1-mock or pcDNA3.1-SNAI2 in U87 cells which was detected by qRT-PCR. **P < 0.001.

Supplementary Table S1: EMT marker genes differentially expressed in U87AR vs. parental U87 cells

**Gene**	**Fold Change (log2)**
Epithelial markers down-regulated in U87AR cells	
CDH11	-3.37
CLDN19	-3.01
LAMC1	-1.43
COL4A3BP	-1.23
TJP2	-1.16
Mesenchymal markers up-regulated in U87AR cells	
SMAD3	3.17
CDH2	3.06
ITGB6	1.94
SNAI2	1.83
TGFBRAP1	0.74
FN1	0.48

Supplementary Table S2: The sequence of the RNA oligoribonucleotides used in miR-203 transfection experiments

**RNA oligoribonucleotides**	**Sequence**
miR-203 mimic	5'-GUGAAAUGUUUAGGACCACUAG-3'
	5'-AGUGGUCCUAAACAUUUCACUU-3'
miR-203 mimic NC	5'-UUCUCCGAACGUGUCACGUTT-3'
	5'-ACGUGACACGUUCGGAGAATT-3'
miR-203 inhibitor	5'-CUAGUGGUCCUAAACAUUUCAC-3'
miR-203 inhibitor NC	5'-CAGUACUUUUGUGUAGUACAA-3'

Supplementary Table S3: Representative gene primers used to perform quantitative polymerase chain reaction (qPCR) of mRNA

**Gene**	**Primer sequences**
SNAI2	Forward: 5'-TGGTTGCTTCAAGGACACAT-3'
	Reverse: 5'-GTTGCAGTGAGGGCAAGAA-3'
ZEB1	Forward: 5'-GGGAGGAGCAGTGAAAGAGA-3'
	Reverse: 5'-TTTCTTGCCCTTCCTTTCTG-3'
E-cadherin	Forward: 5'-CCCGGGACAACGTTTATTAC-3'
	Reverse: 5'-GCTGGCTCAAGTCAAAGTCC-3'
Vimentin	Forward: 5'-CCCTCACCTGTGAAGTGGAT-3'
	Reverse: 5'-TCCAGCAGCTTCCTGTAGGT-3'
GAPDH	Forward: 5'-GAGGTGATAGCATTGCTTTCG-3'
	Reverse: 5'-CAAGTCAGTGTACAGGTAAGC-3'
miR-203	Order from GenePharma: Lot No. 91126N22
U6	Order from GenePharma: Lot No. 8300871042
